# Needle and syringe programs in Yunnan, China yield health and financial return

**DOI:** 10.1186/1471-2458-11-250

**Published:** 2011-04-21

**Authors:** Lei Zhang, Lorraine Yap, Zhuang Xun, Zunyou Wu, David P Wilson

**Affiliations:** 1 National Centre in HIV Epidemiology and Clinical Research, The University of New South Wales, Sydney, Australia; 2 School of Public Health and Community Medicine, The University of New South Wales, Sydney, Australia; 3 National Center for AIDS/STD Control and Prevention, Chinese Center for Disease Control and Prevention, Changping District, Beijing 102206, China; 4 Nantong University, Nantong, China

**Keywords:** HIV, injecting drug users, Yunnan, China, needle-syringe programs, mathematical model, health economics

## Abstract

**Background:**

As a harm reduction strategy in response to HIV epidemics needle and syringes programs (NSPs) were initiated throughout China in 2002. The effectiveness of NSPs in reducing the spread of infection in such an established epidemic is unknown. In this study we use data from Yunnan province, the province most affected by HIV in China, to (1) estimate the population benefits in terms of infections prevented due to the programs; (2) calculate the cost-effectiveness of NSPs.

**Methods:**

We developed a mathematical transmission model, informed by detailed behavioral and program data, which accurately reflected the unique HIV epidemiology among Yunnan injecting drug users (IDUs) in the presence of NSPs. We then used the model to estimate the likely epidemiological and clinical outcomes without NSPs and conducted a health economics analysis to determine the cost-effectiveness of the program.

**Results:**

It is estimated that NSPs in Yunnan have averted approximately 16-20% (5,200-7,500 infections) of the expected HIV cases since 2002 and led to gains of 1,300-1,900 DALYs. The total $1.04 million spending on NSPs from 2002 to 2008 has resulted in an estimated cost-saving over this period of $1.38-$1.97 million due to the prevention of HIV and the associated costs of care and management.

**Conclusion:**

NSPs are not only cost-effective but cost-saving in Yunnan. Significant scale-up of NSPs interventions across China and removal of the societal and political barriers that compromise the effects of NSPs should be a health priority of the Chinese government.

## Background

HIV epidemics in Asia were initially driven by injecting drug use and sex work. Waves of infection have historically occurred in these population groups, followed by infection among clients of sex workers and their regular sexual partners [1,2]. The first recognizable HIV outbreak in China occurred among injecting drug users (IDUs) in the city of Ruili, Yunnan province in 1989 [3], following which the epidemic rapidly expanded throughout Yunnan and neighboring provinces. By 2009, an estimated 740,000 people were infected with HIV in China, including 105,000 with AIDS [4]. Yunnan, a multi-ethnic province of China with a long history of opium and heroin trade and high prevalence of illicit drugs [5], have accounted for over one-quarter of all HIV cases in China [6,7]. HIV in Yunnan has primarily spread through intravenous drug use with a high annual incidence rate of 2.2%-8.0% [8]. These intravenous drug users are young and approximately 80% of them are in their 20 s and 30 s [9]. In 2002, over a decade after the epidemic commenced, needle and syringes programs (NSPs) were initiated by various agencies throughout China as a harm reduction strategy. Currently it is estimated that more than 100 needle and syringe exchange sites are operating throughout Yunnan province alone and a total of 2.5 million syringes were distributed in 2008 [10,11]. Despite the large investment in NSPs, it is estimated that less than 25% of IDUs in Yunnan obtain their injecting equipment through NSPs [12,13] and rates of sharing of injecting equipment remains as high as 45% [14]. NSPs have been shown to be a safe and effective means to reduce HIV transmission in some developed and developing country settings [15-20]. Therefore, it is important to investigate whether the same degree of effectiveness has been achieved in mitigating the spread of infection in a setting such as China, with an established HIV epidemic among IDUs.

In this study we aim to (1) estimate the population benefits that NSPs in Yunnan have likely had in preventing HIV infections and related health outcomes among IDUs; (2) calculate the cost-effectiveness of NSPs from a governmental perspective. We estimate the expected number of infections averted due to NSPs through the development of a mathematical transmission model. The model uses data on the numbers of units of injecting equipment distributed by Yunnan NSPs and behavioral and clinical data from Yunnan province (such as rates of needle sharing between IDUs, rates of disposal of used needles, frequency of injecting, natural history of HIV disease progression in infected IDUs, initiation of antiretroviral therapy), coupled with biological data from the international literature and local HIV epidemiological data. The model is calibrated to accurately reflect the unique HIV epidemiology (incidence, prevalence and aggregate reported number of relevant clinical outcomes including deaths) among Yunnan IDUs and then it is used to estimate the likely epidemiological and clinical outcomes if NSPs were not present. We also conduct a health economics analysis by comparing the financial costs associated with implementing NSPs with the costs saved due to averting infections to determine how cost-effective the program has been from a societal perspective. We consider the cost-effectiveness over the last 7 years and also lifetime value of the program.

## Method

**Figure 1:**
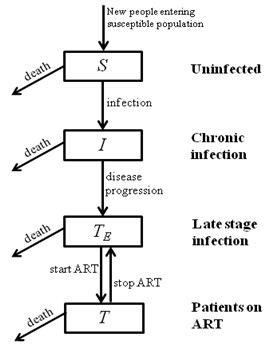
**Schematic diagram of the flow between health states described in the mathematical model**.

A mathematical transmission model was developed to describe the HIV epidemic among IDUs in Yunnan. The model is based on a system of ordinary differential equations [21] and is used to track the rates of HIV transmission from infected IDUs to susceptible IDUs through injecting-related risk behavior and the natural history of infection for HIV-infected individuals as presented in the schematic diagram in Figure 1. This is well-developed epidemiological model that has been used in numerous occasions and has been shown to accurately reflect the dynamic of HIV epidemic [21]. This standard schematic diagram is mathematically translated into 4 ordinary differential equations, one equation for each health state. The health states represented in the model are: uninfected and potentially susceptible individuals (*S*), and HIV-infected individuals that are either in the asymptomatic chronic stage (*I*), treatment-eligible stage (CD4 count < 200 cells/μl) (*T_E_*) or are receiving antiretroviral treatment (*T*). The differential equations to describe the change in the number of people in each of these disease states are:(1)(2)(3)(4)

As such, the model tracks the numbers of HIV-infected people as they progress in disease through the long chronic/asymptomatic infection to late-stage infection; treatment-eligibility in China is based on progression to late-stage infection (CD4 count < 200 cells/μl [22]) and this is what is described in this model. The rates of disease progression are assumed to be constant over time and represent population average rates. Effective antiretroviral therapy (ART) is assumed to reduce viral load in treated individuals and consequently decrease the likelihood of transmitting the virus (by 95% on average, assuming the same relative reduction in infectiousness as in other modes of transmission [23]).

The number of IDUs in the population is defined as *N *and it is assumed that each IDU injects an average of *n *times per year. A proportion, *s*, of all IDUs may share their syringes with others and they do so in a proportion, *q*, of their injections. It is assumed that if sharing of injecting equipment occurs then it happens between two IDUs. The probability of infection from a contaminated syringe per use is denoted as *β_I_*, but people who are on treatment are expected to have a lower probability of transmission *β_T _*due to suppressed viral load [23]. In the model, syringe cleaning has effectiveness *ε_C _*and cleaning occurs before an average proportion of *p_C _*shared injections. Given these definitions, the proportion of sharing events in total per person per year is *nsq*/2 and each susceptible person could acquire infection with probability (1 - *p_C_ε_C_) β_I _*or (1 - *p_C_ε_C_)β_I _*depending on the HIV stage of his/her injecting partner. Therefore, considering the proportion of the entire population in each disease stage, the force of infection *λ*, which estimates the average per-capita risk of infection [21], can then be calculated as(5)

Official figures over the past decade indicate that the number of registered drug users in Yunnan varies between 50,000-70,000, among which ~55% are IDUs [14,24,25]. The actual number of IDUs may be much higher as it is widely accepted that in China there are approximately 4 implicit IDUs behind every registered IDU [26,27]. In this study a sensitivity analysis is carried out on the IDU population size such that the number of IDUs is (i) 2.5 times the registered number; or (ii) 4 times the registered number.

IDUs in Yunnan may obtain syringes through both commercial means and NSPs, but the majority (~75-95%) are obtained commercially [12,13]). Therefore, we assume that the majority of NSP-based distribution of sterile needles and syringes adds to the total numbers that are in circulation. If *P_M _*and *P_N _*commercial and NSP syringes are distributed each year through these means, respectively, and a proportion *ω *of all syringes are not used, then the number of syringes distributed that are used is (*P_M _*+ *P_N_*)(1 - *ω*). In this model it is assumed that syringe distribution by NSPs (*P_N_*) increases linearly since its initiation but commercial syringe distribution (*P_M_*) remained constant throughout these years. If a non-shared needle is used *δ_p _*times on average before disposal and a shared needle is used an average of *δ_s _*times before disposal then(6)

defines a relationship between the total number of needles distributed and their use. This relationship was defined previously by Kwon et al [15]. Several factors may change due to changes in the number of needles distributed. These include: the proportion of syringes that remain unused (*ω*), the proportion of IDUs who share injecting equipment (*s*) and the prevalence of sharing injecting equipment (*q*), or the average number of times each syringe is used before it is disposed (*δ_p_, δ_s_*). Changes in *ω *and *δ_p _*will not influence transmission levels of the virus but changes in *s, q *and *δ_s _*could potentially result in significant changes in HIV incidence. Previous studies have demonstrated that the average number of times a syringe is used before it is disposed varies very little [28-31] and that IDUs are unlikely to share if they have access to clean injecting equipment [32]; therefore, it is assumed that sharing rates (*q*) change with syringe distribution (according to equation (6)) and other values remain constant. Parameter estimates used in the model are presented in Table 1.

**Table 1:** Input values for the mathematical model

Parameter	Description		Values	References
**Biological transmission parameters**				

*β*	Probability of HIV transmission per injection with a contaminated syringe		0.001 - 0.005	[50-57]

*η*	Rate of disease progression from chronic infection to treatment-eligible stage		11-15%	[58]

*μ_c_*	Death rate for HIV-infected people in chronic stage		0.06-0.11%	[59,60]

*μ_a_*	Death rate for HIV-infected people in treatment-eligible stage		3.00-7.86%	[59,60]

*μ_t_*	Death rate for people on ART		0.06-0.11%	[59,60]

*Τ*	Rate of diagnosed people on AIDS stage initiating treatment		0-10%	estimated *^a^*

σ	Rate of people on ART stopping treatment		15-20%	[61,62]

**Epidemiology and NSP parameters**				

*p(t*)	Prevalence among IDUs in Yunnan province		20-30%	[9]

*N*	Population size of IDUs in Yunnan province	SF = 2.5	95,000-125,000	estimated *^b^*
			
		SF = 4.0	150,000-200,000	

*π*	Rate of new entrants into the IDU population		6000	estimated *^c^*

*P_n_*	Total number of syringes distributed through NSP per year (2002-2008)		8.75 × 10^6^	personal communication *^d^*

*R*	Percentage of syringes obtained through NSPs		5-25%	[12,13]

*ω*	Percentage of syringes distributed that are not used		0.5-1%	Assumption

**Behavioral parameters**				

*n*	Average frequency of injecting per IDU per year (weighted average of daily and non-daily injectors)		300-800	[12,31,63-65]

*s*	Proportion of IDUs who share syringes		40-90%	[12,14,31,66-69]

*q*	Proportion of injections that are shared for IDUs that share syringes		29.4%	[66]

*δ_t_*	Average number of times each syringe is used before disposal		2-4	[28-31]

*δ_p_*	Average number of times each non-shared syringe is used before disposal		1-3	estimated *^e^*

*δ_s_*	Average number of times each shared syringe is used before disposal		3-15	estimated *^e^*

**Syringe cleaning parameters**				

*P_c_*	Proportion of syringes used multiple times by multiple people that are cleaned before re-use		20-40%	[12,70]

*ε_c_*	Effectiveness of syringe cleaning		70-80%	[71,72]

a. This number is estimated by dividing the number of HIV-infected IDUs initiating ART each year by the total number of IDUs who live with HIV. It is important to note that ART is only initiated after 2004, hence τ = 0 prior to 2004. We estimated that approximately 10% IDUs are on ART in 2008 and assumed a linear growth of percentage between 2004 and 2008.

b. In Yunnan, official figures indicate that the number of registered drug users varies between 50,000 to 70,000 in the last decade [73-75]. Among these registered drug users, 55% are intravenous drug users period [14,24,25], which corresponds to an increase of ~30,000 to ~40,000 registered IDUs in the last decade. It is widely accepted that in China behind every registered IDUs there is about 2.5-4.0 implicit IDUs that are unregistered [26,27]. The total number of IDUs in Yunnan is estimated to lie between 95,000 and 125,000 if the scaling factor equals to 2.5, whereas the population size is between 150,000 and 200,000 if scaling factor equals to 4.

c. *The entrance rate of IDUs is calculated from the variation of population size, whose minimum and maximum bounds are estimated to be 30,000 and 50,000 over the period 2002-2008 based on the above scenarios. Therefore, an average entrance rate is estimated to be 6000*.

d. The cumulative number of syringes distributed in Yunnan during the period 2002-2008 is approximately 875,000. This number is obtained through personal communication with stakeholders from China CDC.

e. Given that the average usage of a syringe (*δ_t_*) among Yunnan IDUs is about 3 [28-31], the average usage of a non-shared syringe (*δ_P_*) is assumed to be less than 3 and greater than 1. Therefore, the average usage of a shared syringe (*δ_S_*) can then be estimated to be 3-15 by equation *δ*_*t *_= *s*·*q*·*δ*_*s *_+ (1 - *sq*)·*δ*_*P*_.

The model was solved in Matlab and numerical solutions yielded estimates of: the annual number of new HIV incident cases, the number of treatment-eligible HIV patients, the number of people on ART, the number of HIV/AIDS-related deaths, and the total size of the HIV-infected population under conditions of actual distribution of needles and syringes by NSPs and predicted estimates if NSPs had not existed. The model was calibrated to reflect available epidemiological data, mainly HIV prevalence. The calibration was performed by adjusting biological and epidemiological parameters inside the model within their corresponding uncertainty bounds until the model-generated HIV prevalence curve closely mimics the trend of actual data. Retrospectively the model was then used to estimate the expected trajectories of the HIV epidemic in China in the absence of NSPs under the assumption that the reduced number of units of injecting equipment affected sharing rates (according to the supply-demand relationship of equation 6). We estimated the number of disability-adjusted life years (DALYs) gained, and number of HIV cases and deaths averted due to NSPs in Yunnan.

A health economics analysis was also carried out to estimate the cost-effectiveness of the programs for both periods of 2002-2008 and lifetime of IDUs. The lifetime economic impacts of NSPs are evaluated by summing the future costs of currently survived HIV-infected IDUs beyond 2008, without consideration of new future infections. The epidemiological model results became inputs in the health economic analysis. The amount of money invested in NSPs was compared with the estimated costs saved due to averting infections that would have resulted in expenses for monitoring, care, treatment. The costs of NSPs during the period 2002-2008 is calculated by multiplying the average unit expense of distributing a syringe ($0.11 USD), which incorporates and averages over all necessary infrastructure, personnel, marketing and recurring service costs [33], to the estimated total number of syringes distributed. A conservative approach was taken with the inclusion of costs related to routine HIV tests, viral load and CD4 testing and any antiretroviral treatment programs that were supported or subsidized by the Chinese government under its "four free one care" policy [34]. The costs of the programs were calculated by multiplying the unit price of service items, which are determined from published literature and policy documents, to the quantities of provision (Table 2). All costs associated with program investment, infection, treatment and care are listed in Table 2. Undiscounted and 3% discounting analyses were performed.

**Table 2:** Summary of economic results

		2002-2008			2002-Lifetime		
**Cumulative number of cases**		**W/o NSPs**	**With NSPs**	**Averted**	**W/o NSPs**	**With NSPs**	**Averted**

DALYs^a^	(SF = 2.5)	44,391	43,007	1,384	128,879	116,126	12,753
	(SF = 4.0)	76,606	74,628	1,978	225,854	207,582	18,272
HIV incidence	(SF = 2.5)	25,975	20,712	5,263	25,975	20,712	5,263
	(SF = 4.0)	45,511	37,970	7,541	45,511	37,970	7,541
Number of total infected patients in 2008	(SF = 2.5)	35,741	30,998	4,743	35,741	30,998	4,743
	(SF = 4.0)	63,111	56,313	6,797	63,111	56,313	6,797
Number of patients on ART in 2008	(SF = 2.5)	1,800	1,739	61	1,800	1,739	61
	(SF = 4.0)	3,107	3,020	87	3,107	3,020	87
Number of TE patients (person-years)	(SF = 2.5)	58,664	57,815	849	174,166	158,819	15,347
	(SF = 4.0)	100,675	99,462	1,213	304,298	282,308	21,990

Number of patients on ART (person-years)	(SF = 2.5)	3,083	3,025	58	68,004	60,483	7,522
	(SF = 4.0)	5,307	5,224	83	119,487	108,710	10,777

**Governmental investment (millions, in 2009 dollars with 3% discount)**							

Total NSP investment^b^		--	$1.04 m	--	--	$1.04 m	--

Expenses stratified by service items							
Viral load tests^c^	(SF = 2.5)	$1.03 m	$1.01 m	$0.02 m	$15.43 m	$13.86 m	$1.57 m
	(SF = 4.0)	$1.77 m	$1.74 m	$0.03 m	$27.06 m	$24.81 m	$2.25 m
CD4 load tests^d^	(SF = 2.5)	$21.96 m	$21.10 m	$0.85 m	$50.80 m	$45.97 m	$4.83 m
	(SF = 4.0)	$38.00 m	$36.78 m	$1.22 m	$88.99 m	$82.07 m	$6.92 m
Provision of ART^e^	(SF = 2.5)	$10.29 m	$10.09 m	$0.19 m	$154.31 m	$138.57 m	$15.74 m
	(SF = 4.0)	$17.71 m	$17.43 m	$0.27 m	$270.60 m	$248.05 m	$22.55 m
Subsidies on Treatment of OIs^f^	(SF = 2.5)	$26.40 m	$26.04 m	$0.36 m	$61.77 m	$57.14 m	$4.63 m
	(SF = 4.0)	$45.29 m	$44.77 m	$0.51 m	$107.59 m	$100.95 m	$6.63 m
Subsidies on Chinese herbal treatment^g^	(SF = 2.5)	$1.24 m	$1.23 m	$0.02 m	$2.91 m	$2.69 m	$0.22 m
	(SF = 4.0)	$2.13 m	$2.11 m	$0.02 m	$5.06 m	$4.75 m	$0.31 m

Total expenses associated with infection	(SF = 2.5)	$60.91 m	$59.47 m	$1.44 m	$285.22 m	$258.23 m	$26.99 m
	(SF = 4.0)	$104.89 m	$102.83 m	$2.06 m	$499.30 m	$460.63 m	$38.66 m

Expenses stratified by target groups							
Expenses on HIV asymptomatic patients^h^	(SF = 2.5)	$14.86 m	$14.11 m	$0.75 m	$26.90 m	$24.09 m	$2.80 m
	(SF = 4.0)	$25.82 m	$24.74 m	$1.08 m	$47.26 m	$43.24 m	$4.02 m
Expenses on AIDS patients^i^	(SF = 2.5)	$34.16 m	$33.70 m	$0.46 m	$79.94 m	$73.95 m	$5.99 m
	(SF = 4.0)	$58.61 m	$57.94 m	$0.66 m	$139.23 m	$130.65 m	$8.58 m
Expenses on AIDS patients on ART^j^	(SF = 2.5)	$11.89 m	$11.67 m	$0.22 m	$178.38 m	$160.19 m	$18.19 m
	(SF = 4.0)	$20.47 m	$20.15 m	$0.32 m	$312.81 m	$286.75 m	$26.06 m

Total expenses associated with infection	(SF = 2.5)	$60.91 m	$59.47 m	$1.44 m	$285.22 m	$258.23 m	$26.99 m
	(SF = 4.0)	$104.89 m	$102.83 m	$2.06 m	$499.30 m	$460.63 m	$38.66 m

**Cost Benefit Analysis**							
Cost/DALY averted	(SF = 2.5)		$753			$82	
	(SF = 4.0)		$527			$57	
Benefit-cost ratio (ratio of expenses saved to investment)	(SF = 2.5)		1.38			25.89	
	(SF = 4.0)		1.97			37.09	
Cost/Infection averted	(SF = 2.5)		$198			$198	
	(SF = 4.0)		$138			$138	

a. The cumulative numbers of DALYs are calculated based on the values of health utilities at different disease stages. Health utilities among asymptomatic people living HIV, people at AIDS stage and patients receiving ART are 0.88 (0.82-0.94) [76,77], 0.64 (0.58-0.70) [77] and 0.78 (0.76-0.80) [76,78] respectively.,

b. The costs of NSPs during the period 2002-2008 is calculated by multiplying the average unit expense of distributing a syringe ($0.11 USD), which incorporates and averages over all necessary infrastructure, personnel, marketing and recurring service costs [33], to the estimated total number of syringes distributed (8.75 × 10^6^).

c. Regular viral load monitoring is currently undertaken once a year for HIV/AIDS patients on ART to monitor potential change in viral load. Its cost is hence the product of the number of patients on ART and its unit cost (~USD$300/person [79,80]) and is calculated with 3% value discounting.

d. CD4 load tests are performed quarterly for patients on ART [80-82] and twice a year for people diagnosed with HIV but not on ART [80,82]. The unit cost of CD4 load test is USD $42 [83]. The total cost is calculated with 3% discounting.

e. The annual cost of ART for each AIDS patient is approximately USD $3000 [83-86], which is multiplied by the number of patients on ART to obtain the total cost. The total cost is calculated with and without value discounting.

f. Each year, the government subsidizes approximately USD $340 [87] of healthcare associated with opportunistic infections of symptomatic AIDS patients (those that are in the treatment-eligible stage or potentially on ART (but experienced treatment failure) in our model). Its total yearly spending is calculated as the product of the two with 3% value discounting.

g. Each year, the government subsidizes approximately USD $16 [87] of herbal treatment for each symptomatic AIDS patient (treatment-eligible patients and patients on ART). Its total yearly spending is calculated as the product of the two with and without value discounting.

h. Each HIV asymptomatic patients are CD4 tested twice a year [80,82], the governmental investment on each individual HIV patients is USD$42 × 2 = USD$84. This amount is multiplied by the total number of asymptomatic patients and then summed with the cost of one-off diagnosis testing of newly infected cases to result in the total governmental investment in this specific target group.

i. Each HIV-infected patient yet to receive ART will receive CD4 testing twice each year [80,82]. Treatment of opportunistic infections and Chinese herbal treatment for AIDS are also covered or subsidized by the government [87].

Each patient on ART will receive 1 viral test and 4 CD4 tests each year [79-82]. In addition to government-subsidized treatment for opportunistic infections and herbal treatment for AIDS, they also receive ART on governmental expenses [85].

## Results

**Figure 2:**
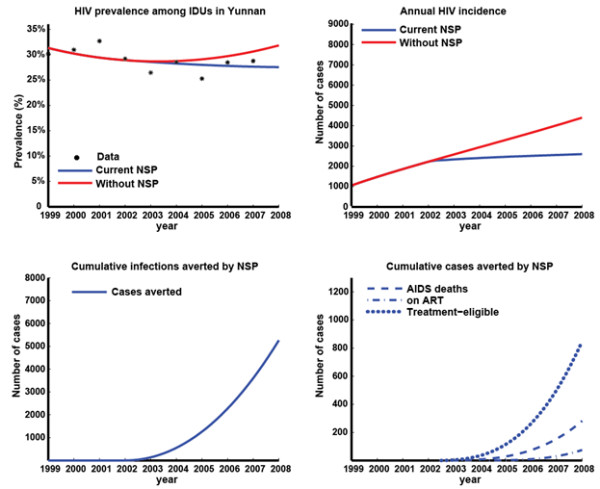
**Results of epidemiological model with IDU population size scaling factor 2.5 times the registered number of IDUs**. **(a)** Extracted published prevalence data [9] and model-based estimates of HIV prevalence among IDUs in Yunnan with and without NSPs; **(b) **model-based estimates of HIV incidence among IDUs in Yunnan with and without NSPs; **(c) **Estimated cumulative number of HIV infections averted due to NSPs; **(d) **Estimated cumulative number of AIDS deaths, people on ART, and people in treatment-eligible stage averted due to NSPs.

**Figure 3:**
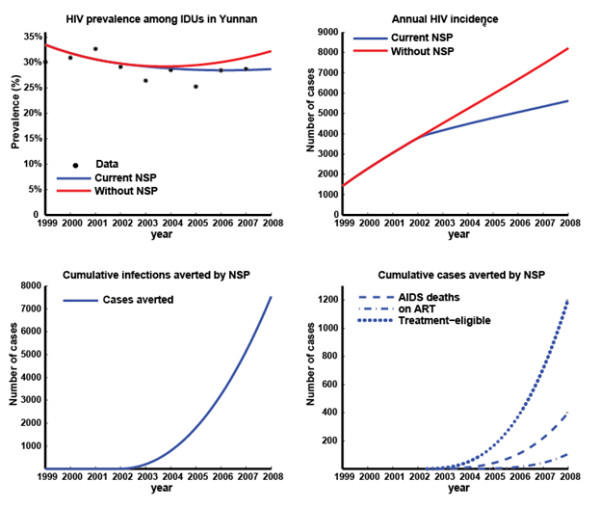
**Results of epidemiological model with IDU population size scaling factor 4.0 times the registered number of IDUs**. **(a) **Extracted published prevalence data [9] and model-based estimates of HIV prevalence among IDUs in Yunnan with and without NSPs; **(b) **model-based estimates of HIV incidence among IDUs in Yunnan with and without NSPs; **(c) **Estimated cumulative number of HIV infections averted due to NSPs; **(d) **Estimated cumulative number of AIDS deaths, people on ART, and people in treatment-eligible stage averted due to NSPs.

The epidemiological model accurately resembles the epidemiological trends of HIV prevalence in Yunnan during the past decade (Figure 2, 3). The model estimates that according to scaling factor (SF) assumptions of the total IDU population size being 2.5-times (Figure 2) or 4-times (Figure 3) the number of registered IDUs, approximately 20,712 or 37,970 new HIV infections have occurred among IDUs during the period of NSPs, respectively. The model also estimated that NSPs have averted 5,263 (SF = 2.5; 7,541 for SF = 4) new HIV infections which accounts for 20% (SF = 2.5; 16% for SF = 4) of infections without NSPs and resulted in a gain of 1,384 (SF = 2.5; 1,978 for SF = 4) DALYs, and will gain a further 12,753 (SF = 2.5; 18,272 for SF = 4) DALYs in the patients' lifetimes. In addition, an extra 849 (SF = 2.5; 1,213 for SF = 4) person-years in the AIDS stage have likely been averted by NSPs and a further 15,347 (SF = 2.5; 21,990 for SF = 4) person-years will be saved in the patients' lifetimes due to the lasting effects of the implemented program. NSPs have led to an estimated 41% (SF = 2.5; 32% for SF = 4) decline in annual HIV incidence in 2008.

Since the initiation of NSPs in Yunnan in 2002, about 875,000 syringes were distributed throughout the province, costing USD$1.04 m. The total expenditure for the care, management and treatment of HIV-infected people since 2002 was USD$59.47 m (SF = 2.5; USD$102.83 m for SF = 4) (3% discounting), of which 56% were spent on treatment-eligible patients who are not yet receiving antiretroviral therapy (ART), whereas the remainder was spent on asymptomatic patients (24%) and those on ART (20%). However, if NSPs did not exist, the model estimates that the Chinese government would have spent an extra USD$1.44 m (SF = 2.5; USD$2.06 m for SF = 4) on HIV/AIDS patients care and management over the past decade due to the higher HIV incidence. Since this saving of healthcare expenses is greater than the initial investment, these programs are not only very cost-effective but cost-saving even over this short time period. The investment in NSPs in Yunnan since 2002 will yield even greater savings in the long term. In the lifetime of the current population, the benefits of averting infections over the last 10 years will lead to large future savings, of USD$27 m (SF = 2.5; $39 m for SF = 4) (3% discounting) over the population lifetime.

The return on investment of the NSPs in Yunnan was also assessed with a cost-utility analysis. It is estimated that the cost for each DALY gained since 2002 is approximately USD$753 (SF = 2.5; USD$527 for SF = 4), but the value reduces to USD$82 (SF = 2.5; USD$57 for SF = 4) if the lifetime impacts of the program are considered. Healthcare programs are generally considered cost-effective if the cost per DALY saved is less than the per capita gross national income (GNI) [35]. The Chinese per-capita GNI is USD$3,650 in 2009 [36]. Additionally, with an estimate of NSP investment 1.04 million, the directly gained economic benefit is measured to be 1.4-2.0 million during 2002-2008, which corresponds to a benefit-cost ratio of 1.38-1.97 (SF = 2.5, 4 respectively, Table 2). Thus, this program is extremely cost-effective compared to most healthcare programs. In fact, not only are NSPs in Yunnan cost-effective, but we have calculated that they are actually cost-saving. That is, the return in healthcare savings is greater than the initial investment. Specifically, for every one dollar the government invested into NSPs, it was estimated the government would gain back that dollar plus an additional USD$0.38 (SF = 2.5; USD$0.97 for SF = 4) due to the reduced number of HIV-related tests and treatments. The return on investment is even greater when the lifetime effects of these programs are taken into account: ~USD$15 (SF = 2.5; USD$36 for SF = 4) are returned for every USD$1 invested over the past 7 years. We also estimated that it cost USD$198 (SF = 2.5; USD$138 for SF = 4) of investment into NSPs to avert a new HIV infection.

## Discussion

We estimated that the spending of a total USD$1.04 million on NSPs from 2002 to 2008 has resulted in cost-savings of USD$1.38-1.97 million due to the prevention of HIV and the associated costs of care and management. During the same time period more than 1,300-1,900 DALYs and more than 5,200-7,500 infections have been saved. Lifetime projections suggest that continued substantial savings of costs and gains in years of life would occur. Even though there is an established HIV epidemic and HIV transmission has likely continued at moderately high levels, NSPs in Yunnan are still shown to be very effective in reducing the total number of HIV transmissions among IDUs, averting approximately 16-20% of the potential HIV cases since 2002. This is comparable to a similar investigation carried out in 2006 in Ukraine over its one year harm reduction intervention, which consequently lead to a 22% decrease in IDU HIV incidence and 1% decrease in prevalence [16]. IDU-initiated HIV epidemics remain as confined epidemics and have not yet generalized to other populations in China.

The current study is an important study that evaluated the cost-effectiveness of NSPs for IDUs in China. We have shown the programs to be cost-saving. The fact that the benefit-cost ratio increases substantially over the lifetime of the population reflects the long-term impacts of the programs. Our estimate of USD$138-198 in spending per infection averted is consistent with similar studies in other locations [16,37,38]. By adjusting the cost to account for the purchasing power parity for China (2007 PPP factor was 4.09 relative to 1 USD), the cost translates to $560-810 per infection averted, which is still very cost-effective in comparison to many developed countries ($3,000-$20,000 [39-42]).

Our analysis involved a conservative calculation of the cost-effectiveness of NSPs by only including governmental investment in HIV/AIDS alone. The actual savings will be much higher for a number of reasons. First, hepatitis C virus (HCV) has a very high prevalence among Chinese IDUs (55-80% [43,44]). A 2009 report on the cost-effectiveness of Australian NSPs demonstrated that the majority of savings by NSPs are actually due to HCV-related healthcare expenses saved and not HIV [45]. Second, other medical costs such as those due to mental health episodes, injecting related injury, psychosocial benefits, overdose education and prevention may further increase the return on investment. Third, we did not include patient/client costs or productivity losses or gains. AIDS patients are contributing a substantial out-of-pocket payment while accessing government-supported services, including transportation to clinic/hospital, consultation fees and hospitalization expenses. Therefore, our calculations should be regarded as a very conservative estimate of the actual expenses saved.

Several limitations of the current study should be noted. First, the cost data used in this study was collected from a number of sources from Yunnan and also other provinces [39-45]. Treatment-related cost variations may occur due to differences in economic development in different provinces. However, these differences are considered to be small as the treatment prices are standardized in governmental health institutions under the national "four free one care" policy. Second, the current study is based on ART eligibility at a CD4 threshold of 200 cells/μl. China has recently adopted a new guideline to initiate treatment at 350 cells/μl [46]. This will likely significantly increase the potential medical expenses for ART patients in their lifetime. Besides, the current study does not take into consideration of secondary impacts of NSPs, e.g. reduction of infections among sexual partners of IDUs due to decrease of HIV prevalence among IDUs as a result of NSPs. Therefore, our analysis should be regarded as a conservative estimate of the actual cost-effectiveness of NSPs in Yunnan. Third, this study used a simple deterministic approach and did not include a detailed uncertainty analysis. However, we took into consideration the variation in IDU population size, which represents the largest and most important uncertain parameter. Results from this analysis should be regarded as an estimation of the average effectiveness and cost-effectiveness of NSPs in Yunnan. Finally, a notable limitation of the current epidemiological model is that it does not include the quitting rate of IDUs, which may vary the population size substantially. We assume that an IDU leaves the population only through death.

The current coverage of NSPs in the province remains low, as less than 25% of IDUs access NSPs. This suggests that it is possible to have even greater health and economic gains by expanding the programs further in the future. Large changes in government attitudes occurred from viewing drug users as criminals to large-scale adoption of needle and syringe provision [47]. Ongoing police raids and confinement of drug users cause increasing fear of police arrest and reluctance to access to sterile equipment from NSP sites or peer educators. Compulsory detention of IDUs in China in detoxification centers and labor camps largely limits the accessibility of IDUs to NSPs [48,49]. Hence, enhancing cooperation between multiple institutions including the Ministry of Health and Public Security and Justice is necessary for further scale-up of the programs. Other issues include reducing social stigma against drug users and maintaining users' anonymity in NSP sites. If such barriers can be removed and NSPs expanded then there is strong potential for the large epidemiological and economic benefits to increase even more substantially. Whilst these results are specific to Yunnan, China, the qualitative conclusions are generally applicable to other settings that are considering commencing or expanding NSPs where HIV is endemic among IDUs. NSPs not only save lives and health outcomes but they are also a valuable economic investment.

## Conclusion

Conclusively, NSPs are not only cost-effective but cost-saving in Yunnan since the implementation of the programs and will have greater epidemiological and economic benefits if in the life span of IDUs. Significant scale-up of NSPs interventions across China and removal of the societal and political barriers that compromise the effects of NSPs should be a health priority of the Chinese government.

## Competing interests

The authors declare that they have no competing interests.

## Authors' contributions

LY and ZX were responsible for data collection and part of the literature review. LZ conducted the statistical analysis and literature review, and drafted the manuscript. DPW and ZW are the principal investigators, advised on the analytic approaches and assisted in the manuscript writing. All authors read and approved the final version.

## Pre-publication history

The pre-publication history for this paper can be accessed here:

http://www.biomedcentral.com/1471-2458/11/250/prepub

## References

[B1] Ruxrungtham K Brown T Phanuphak P HIV/AIDS in Asia Lancet 2004 364 9428 69 82 10.1016/S0140-6736(04)16593-8 15234860

[B2] Weniger BG The epidemiology of HIV infection and AIDS in Thailand AIDS 1991 5 Suppl 2 S71 85 1845063 10.1097/00002030-199101001-00011

[B3] Ma Y L Z Zhang KL HIV was first discovered among injection drug users in China Chinese Journal of Epidemiology 1990 11 3 184 185

[B4] Lu F Estimating the number of people at risk for and living with HIV in China in 2005: methods and results Sex Transm Infect 2006 82 Suppl 3 iii87 91 16735299 10.1136/sti.2006.020404 PMC2576728

[B5] Chin K l C Zhang SX The Chinese Connection: Cross-border Drug Trafficking between Myanmar and China 2007 U.S. Department of Justice: Newark

[B6] Xiao Y Expansion of HIV/AIDS in China: lessons from Yunnan Province Soc Sci Med 2007 64 3 665 75 10.1016/j.socscimed.2006.09.019 17107739 PMC2730760

[B7] Joint United Nations Programme on HIV/AIDS China Office 2005 China HIV/AIDS Epidemic data 2005 http://www.unaids.org.cn/uploadfiles/20080725142719.pdf cited 2009 October 24

[B8] Lu L The changing face of HIV in China Nature 2008 455 7213 609 11 10.1038/455609a 18833270

[B9] Jia M The HIV epidemic in Yunnan Province, China, 1989-2007 J Acquir Immune Defic Syndr 2010 53 Suppl 1 S34 40 20104107 10.1097/QAI.0b013e3181c7d6ff

[B10] Wang G The report and analysis on Global Fund AIDS Program Round 4 in Kaiyuan County Soft Science of Health 2008 22 2 178 180

[B11] Establishing community-operating needles and syringes exchange sites 2004 http://www.chain.net.cn/wzhg/7590.htm cited 2009 August 25th

[B12] Cheng F SASH survey on high risk behaviors of IDUs in four cities of Yunnan and Sichuan Chinese Journal of Drug Dependence 2003 12 4 294 298

[B13] Sha L A sampling survey on syringe exchange and methadone maintenance treatment among drug abusers in Yunnan province Chinese Journal of AIDS/STD 2008 14 3 238 239

[B14] Chu TX Levy JA Injection drug use and HIV/AIDS transmission in China Cell Res 2005 15 11-12 865 9 10.1038/sj.cr.7290360 16354561

[B15] Kwon JA The impact of needle and syringe programs on HIV and HCV transmissions in injecting drug users in Australia: a model-based analysis J Acquir Immune Defic Syndr 2009 51 4 462 9 10.1097/QAI.0b013e3181a2539a 19387355

[B16] Vickerman P The cost-effectiveness of expanding harm reduction activities for injecting drug users in Odessa, Ukraine Sex Transm Dis 2006 33 10 Suppl S89 102 16735956 10.1097/01.olq.0000221335.80508.fa

[B17] Jenkins C Measuring the impact of needle exchange programs among injecting drug users through the National Behavioural Surveillance in Bangladesh AIDS Educ Prev 2001 13 5 452 61 10.1521/aeap.13.5.452.24141 11718444

[B18] Wodak A Cooney A Do needle syringe programs reduce HIV infection among injecting drug users: a comprehensive review of the international evidence Subst Use Misuse 2006 41 6-7 777 813 10.1080/10826080600669579 16809167

[B19] Bastos FI Strathdee SA Evaluating effectiveness of syringe exchange programmes: current issues and future prospects Soc Sci Med 2000 51 12 1771 82 10.1016/S0277-9536(00)00109-X 11128265

[B20] Wodak A Lessons from the first international review of the evidence for needle syringe programs: the band still plays on Subst Use Misuse 2006 41 6-7 837 9 10.1080/10826080600669587 16809173

[B21] Anderson RM May RM Infectious Diseases of Humans: Dynamics and Control 1991 New York: Oxford University Press

[B22] Zhang F The Chinese free antiretroviral treatment program: challenges and responses AIDS 2007 21 Suppl 8 S143 8 10.1097/01.aids.0000304710.10036.2b 18172383

[B23] Wilson DP Relation between HIV viral load and infectiousness: a model-based analysis Lancet 2008 372 9635 314 20 10.1016/S0140-6736(08)61115-0 18657710

[B24] Yang H Heterosexual transmission of HIV in China: a systematic review of behavioral studies in the past two decades Sex Transm Dis 2005 32 5 270 80 10.1097/01.olq.0000162360.11910.5a 15849527 PMC1791011

[B25] State council AIDS working committee office and UN themem group on HIV/AIDS in China Moh U A Joint Assessment of HIV/AIDS Prevention, Treatment and Care in China 2004 Beijing

[B26] Li B Li C Shi E On enforcement of drug trafficking probihitation between the western China and neighboring countries Journal of Fujian Public Safety College 2004 1 77 14 17

[B27] Du X Analysis and considerations of the current detoxification method in China Chinese Journal of Drug Dependence 2005 14 5 392 398

[B28] Chen L Assessment of results of exchange of syringe among drug users in preventing the transmission of HIV/AIDS China tropical medicine 2007 7 11 2138 2140

[B29] Ming Z Effectiveness of needle exchange combined with peer education among IDUs in Guangxi China Journal of AIDS/STD 2005 11 3 188 191

[B30] Jia Y Predictors of HIV infection and prevalence for syphilis infection among injection drug users in China: Community-based surveys along major drug trafficking routes Harm Reduct J 2008 5 29 10.1186/1477-7517-5-29 18724872 PMC2556669

[B31] Luo J Knowledge of attitude towards HIV/AIDS and risk behavior of 306 drug addicts in Kunming Chinese Journal of Drug Dependence 2002 11 4 300 302

[B32] Bao Y The result analysis on drug users needle syringe comprehensive intervention activities at four counties in Yunnan Chinese Journal of Primary Medications and Pharmaceuticals 2007 14 12 2025 2027

[B33] Masaki E Cost-effectiveness of targeted interventions in GuanaXi, China 2007 http://gametlibrary.worldbank.org/FILES/1416_Guangxi%20Cost%20Effectiveness%20Analysis%202007%20China.pdf cited 2009 Sep 19

[B34] Shao Y AIDS epidemic at age 25 and control efforts in China Retrovirology 2006 3 87 10.1186/1742-4690-3-87 17140434 PMC1702549

[B35] World Health Organization Commission on Macroeconomics and Health. Macroeconomics and Health: Investing in Health for Economic Development 2001 World Health Organization: Geneva

[B36] The World Bank Gross national income per capita 2009, Atlas method and PPP 2009 http://siteresources.worldbank.org/DATASTATISTICS/Resources/GNIPC.pdf cited 2010 16th Feb

[B37] Cabases JM Sanchez E Costs and effectiveness of a syringe distribution and needle exchange program for HIV prevention in a regional setting Eur J Health Econ 2003 4 3 203 8 10.1007/s10198-003-0172-7 15609186

[B38] Kumaranayake L The cost-effectiveness of HIV preventive measures among injecting drug users in Svetlogorsk, Belarus 2004 99 12 1565 76 Addiction 10.1111/j.1360-0443.2004.00899.x 15585048

[B39] Cohen DA Wu SY Farley TA Structural interventions to prevent HIV/sexually transmitted disease: are they cost-effective for women in the southern United States? Sex Transm Dis 2006 33 7 Suppl S46 9 16794555 10.1097/01.olq.0000221015.64056.ee

[B40] Cohen DA Wu SY Farley TA Comparing the cost-effectiveness of HIV prevention interventions J Acquir Immune Defic Syndr 2004 37 3 1404 14 10.1097/01.qai.0000123271.76723.96 15483470

[B41] Laufer FN Cost-effectiveness of syringe exchange as an HIV prevention strategy J Acquir Immune Defic Syndr 2001 28 3 273 8 11694836 10.1097/00042560-200111010-00012

[B42] Harris ZK Efficient allocation of resources to prevent HIV infection among injection drug users: the Prevention Point Philadelphia (PPP) needle exchange program Health Econ 2006 15 2 147 58 10.1002/hec.1021 16145716

[B43] Xia X Epidemiology of hepatitis C virus infection among injection drug users in China: systematic review and meta-analysis Public Health 2008 122 10 990 1003 10.1016/j.puhe.2008.01.014 18486955

[B44] Bao YP Liu ZM Systematic review of HIV and HCV infection among drug users in China Int J STD AIDS 2009 20 6 399 405 10.1258/ijsa.2008.008362 19451325

[B45] Wilson DP Return on investment 2: Evaluating the cost-effectiveness of needle and syringe programs in Australia 2009 Australian Government Department of Health and Ageing: Canberra

[B46] Sun K Recent key advances in human immunodeficiency virus medicine and implications for China AIDS Res Ther 2010 7 12 10.1186/1742-6405-7-12 20500898 PMC2890503

[B47] Hammett TM '*Social evils' and harm reduction: the evolving policy environment for human immunodeficiency virus prevention among injection drug users in China and Vietnam* 2008 103 1 137 45 Addiction 10.1111/j.1360-0443.2007.02053.x 18028519

[B48] Arriola KR Development and implementation of the cross-site evaluation of the CDC/HRSA corrections demonstration project AIDS Educ Prev 2002 14 3 Suppl A 107 18 12092929 10.1521/aeap.14.4.107.23883

[B49] Cohen JE Amon JJ Health and human rights concerns of drug users in detention in Guangxi Province, China PLoS Med 2008 5 12 e234 10.1371/journal.pmed.0050234 19071954 PMC2596857

[B50] Kaplan EH O'Keefe E Let the needles do the talking! Evaluating the New Haven needle exchange Interfaces 1993 23 1 7 26 10.1287/inte.23.1.7

[B51] Hudgens MG Subtype-specific transmission probabilities for human immunodeficiency virus type 1 among injecting drug users in Bangkok, Thailand Am J Epidemiol 2002 155 2 159 68 10.1093/aje/155.2.159 11790680

[B52] Henderson DK Risk for occupational transmission of human immunodeficiency virus type 1 (HIV-1) associated with clinical exposures. A prospective evaluation Ann Intern Med 1990 113 10 740 6 2240876 10.7326/0003-4819-113-10-740

[B53] Cavalcante NJ Risk of health care professionals acquiring HIV infection in Latin America AIDS Care 1991 3 3 311 6 10.1080/09540129108253078 1932195

[B54] Gerberding JL Incidence and prevalence of human immunodeficiency virus, hepatitis B virus, hepatitis C virus, and cytomegalovirus among health care personnel at risk for blood exposure: final report from a longitudinal study J Infect Dis 1994 170 6 1410 7 10.1093/infdis/170.6.1410 7995979

[B55] Ippolito G Puro V De Carli G The risk of occupational human immunodeficiency virus infection in health care workers. Italian Multicenter Study. The Italian Study Group on Occupational Risk of HIV infection Arch Intern Med 1993 153 12 1451 8 10.1001/archinte.153.12.1451 8512436

[B56] Nelsing S Nielsen TL Nielsen JO Occupational exposure to human immunodeficiency virus among health care workers in a Danish hospital J Infect Dis 1994 169 2 478 10.1093/infdis/169.2.478 8106790

[B57] Tokars JI Surveillance of HIV infection and zidovudine use among health care workers after occupational exposure to HIV-infected blood. The CDC Cooperative Needlestick Surveillance Group Ann Intern Med 1993 118 12 913 9 8387737 10.7326/0003-4819-118-12-199306150-00001

[B58] Mellors JW Plasma viral load and CD4+ lymphocytes as prognostic markers of HIV-1 infection Ann Intern Med 1997 126 12 946 54 9182471 10.7326/0003-4819-126-12-199706150-00003

[B59] Ledergerber B Predictors of trend in CD4-positive T-cell count and mortality among HIV-1-infected individuals with virological failure to all three antiretroviral-drug classes Lancet 2004 364 9428 51 62 10.1016/S0140-6736(04)16589-6 15234856

[B60] Smith C Causes of death in D:A:D study-initial results 2008

[B61] Sabin LL Using electronic drug monitor feedback to improve adherence to antiretroviral therapy among HIV-positive patients in China AIDS Behav 2010 14 3 580 9 10.1007/s10461-009-9615-1 19771504 PMC2865631

[B62] Wang H Self-Reported adherence to antiretroviral treatment among HIV-infected people in Central China AIDS Patient Care STDS 2008 22 1 71 80 10.1089/apc.2007.0047 18095837

[B63] Yao Y Sexual behavior and risks for HIV infection and transmission among male injecting drug users in Yunnan, China Int J Infect Dis 2009 13 2 154 61 10.1016/j.ijid.2008.05.1228 18778963 PMC2768780

[B64] Gu J Prevalence of needle sharing, commercial sex behaviors and associated factors in Chinese male and female injecting drug user populations AIDS Care 2009 21 1 31 41 10.1080/09540120802068787 19085218

[B65] Zhao M HIV sexual risk behaviors among injection drug users in Shanghai Drug Alcohol Depend 2006 82 Suppl 1 S43 7 16769445 10.1016/s0376-8716(06)80008-6

[B66] Zhou Z An investigation on high risk behaviors among male drug users with needle sharing South China Journal of Preventive Medicine 2007 33 1 15 18

[B67] Ma X [Survey of behaviors and knowledge about HIV/AIDS among intravenous drug users at a city in Sichuan Province] Sichuan Da Xue Xue Bao Yi Xue Ban 2004 35 3 376 8 15181841

[B68] Liu Z Knowledge and risk behavior on HIV/AIDS among drug addicts in four areas in China Chinese Journal of Drug Dependence 2001 10 1 48 52

[B69] Zheng X Wang X An epidemiology study on HIV transmission through IDU and blood collection or transfusion Chinese Journal of Epidemiology 2003 24 11 1057 1059

[B70] UNAIDS 2008 Report on the global AIDS epidemic 2008 New York

[B71] Abdala N Can HIV-1-Contaminated Syringes Be Disinfected? Implications for Transmission Among Injection Drug Users JAIDS Journal of Acquired Immune Deficiency Syndromes 2001 28 5 487 494 10.1097/00042560-200112150-00013 11744839

[B72] Siegel J Weinstein M Fineberg H Bleach programs for preventing AIDS among iv drug users: modeling the impact of HIV prevalence Am J Public Health 1991 81 10 1273 1279 10.2105/AJPH.81.10.1273 1928525 PMC1405310

[B73] Duan L Ethnic issues and social harmony of bordering regions in Yunnan Journal of the Socialism Institute of Yunnan 2007 1 43-47

[B74] Shen R "ChunHui" Scheme ---- serve for HIV/AIDS control and prevention research in China ShenZhou Scholars 2006 ShenZhou Scholars Press: Beijing 16 17

[B75] Na M Three years people's war against drugs, registered users in Yunnan dropped 25% 2008 http://www.yn.xinhuanet.com/topic/2008-06/25/content_13640496.htm cited 2009 1st Oct

[B76] Commonwealth Department of Health and Ageing Return on investment in needle and syringe programs in Australia 2002 Commonwealth Department of Health and Ageing: Canberra 49

[B77] Tengs TO Lin TH A meta-analysis of utility estimates for HIV/AIDS Med Decis Making 2002 22 6 475 81 10.1177/0272989X02238300 12458977

[B78] Sakthong P Health Utilities in Patients with HIVAIDS in Thailand Value in Health 2009 12 377 384 10.1111/j.1524-4733.2008.00440.x 20667064

[B79] Yang HM Study on the utilization of health services and costs of hospital-based medical care for 29 patients with HIV/AIDS in China Chinese Journal of Epidemiology 2003 24 5 393 6 12820935

[B80] He Q Yuan J Xu Y The Projection of HIV/AIDS Medical Expenses in Guangdong Province Journal for China AIDS/STD 2004 10 4 271 274

[B81] Tang H Analysis of CD4 cell count testing among AIDS patients following antiretroviral treatment in Hubei province Practical Preventive Medicine 2007 14 4 1068 1070

[B82] China CDC National prevention of HIV/AIDS, STIs and HCV in 2009 2009 http://www.bdxcdc.com/jbkz/az/2009-03-12/272.html cited 2009 15th Oct

[B83] Song L The social and economic impact of HIV/AIDS in Ruili county of Yunnan province Department of Public Health 2007 Kuming Medical College: Kuming 61

[B84] Liu K Yuan J Social and economic impacts of HIV/AIDS epidemic in China Journal of Xuehai 2003 5 68 72

[B85] Moon S Out-of-pocket costs of AIDS care in China: are free antiretroviral drugs enough? AIDS Care 2008 20 8 984 94 10.1080/09540120701768446 18777223

[B86] Zhang K.-l The health systems response to HIV in China Journal of Reproductive Medicine 2004 13 6 330 333

[B87] Guo J Socio-economic impacts of HIV/AIDS in Henan province Social medicine and health management 2007 Huazhong University of Science and Technology: Wuhan 199

